# Employees and the National Insurance Act

**Published:** 1912-06-22

**Authors:** 


### NATIONAL INSURANCE ACT DEPARTMENT.

### EMPLOYEES AND THE NATIONAL INSURANCE ACT.

### XVI.—How to Choose an Approved Society.

Government printers have been busy during the past fortnight pouring out leaflets and instructions by the million. The latest leaflet is entitled "N. H. Leaflet," and is to be obtained for the asking at any Post Office. Ten million copies have been printed, and we advise every one of our readers to obtain a copy as soon as possible. The leaflet is the very best thing that has yet been issued, and sets forth concisely all that is really necessary to be known by the insured worker as to his or her position under the Act. We have already dealt in former articles with the benefits and contributions, and there is no necessity for us to repeat this information, but we would strongly emphasise the statement which is twice repeated in this latest leaflet—namely, that the full benefits of Insurance can only be obtained by joining an Approved Society.

The First List of Approved Societies.

This is a most opportune statement in view of the fact that the first list of societies that have obtained the approval of the Insurance Commissioners was published as a White Paper within the past fortnight. This White Paper, which is numbered Cd. 6238, may be obtained through any bookseller or direct from the Government publishers for 2d. There are five main sections in this publication, in which are set forth respectively the societies that have been approved by (1) The Joint Committee of the Insurance Commissioners, (2) The English Commissioners, (3) The Scotch Commissioners, (4) The Irish Commissioners, and (5) The Welsh Commissioners.

The societies approved by the Joint Committee are those which admit members from more than one part of the United Kingdom. Those approved by the separate Commissioners are the societies that restrict their membership to the particular country of the Union. In the first section there are 75 societies, 58 of which admit both men and women, 13 admit men only, and 4 admit women only.

It is also to be noted that two of the societies thus approved are in the nature of a combination of a number of separate organisations. The first of these is the General Federation of Trade Unions, which is composed of no less than 118 distinct societies, and the second is the National Amalgamated Approved Society for National Insurance, which is a combination of 10 Industrial Insurance Companies. It will thus be seen that 201 organisations are already approved for the purposes of the National Insurance Act in such a manner as to admit of their carrying out the duties of approved societies in more than one part of the United Kingdom.

The corresponding figures for the societies included in sections (2) to (5) are as follows:—

| Membership drawn from | Societies Admitting | | | |
|---|---|---|---|---|
| | Men and Women | Men only | Women only | Total |
| 2. England ... | 55 | 22 | 6 | 83 |
| 3. Scotland ... | 21 | 7 | 4 | 32 |
| 4. Ireland ... | 29 | 1 | 1 | 31 |
| 5. Wales ... | 15 | 15 | — | 30 |
| Total... ... | 120 | 45 | 11 | 176 |

In addition to these societies the names of 18 others are given as to which draft rules have been settled with the Commissioners, and, as soon as they have been adopted by the societies concerned, approval will be given.

Combining all the figures it will be found that the first list of approved societies contains the names of 395 separate organisations, so that there would appear to be no lack of opportunity for every insured person to find a society which would be prepared to entertain his or her application for membership.

The Choice of a Society.

It is, perhaps, just as well that so wide an interest is being taken in this matter, because it has been our experience that it is only the active work of canvassing for membership that has aroused the mass of the workers to the advantages of joining an approved society. On the other hand, the large number of societies now in the field is presenting a difficulty in many quarters as to the choice of a particular society. It must be borne in mind in this connection that it is an offence, under the Act, to join or to attempt to join more than one society for the purpose of the National Health Insurance.

Competition is both close and keen in almost all parts of the country. The grounds for selecting one society in preference to another are such as require some consideration. As the immediate advantages are identical, it is largely a question of foresight which should determine the matter of choice.

Sentiment and personal persuasion will frequently be the determining factors, and, while the result of a choice so made may turn out to be correct, we hold that each of our readers should consider the matter independently for himself or herself, and we proceed to set out the essential points that, in our opinion, should receive attention in that consideration.

Points to be Considered.

In the first place, it should be borne in mind that insurance depends upon the operation of the law of averages, and the law of averages cannot be expected to work with any degree of certainty unless it has a wide numerical basis to support it. With large numbers past experience is found to be a most excellent guide to future experience, but with small or comparatively small numbers this is by no means the case. Now the benefits payable under the Act have been regulated according to the contributions on the basis of past sickness experience derived from large numbers of men insured against sickness, and there is no doubt that if all persons who become insured under the Act were to join, or had been required to join, one society, the future experience of that one comprehensive society would almost exactly coincide with the tables employed by the actuaries in framing the estimates. It does not follow, however, that the same table is equally applicable to an isolated society carrying on its operations in a particular area or amongst those following a particular trade or occupation. We do not suggest that the departure from the general table will always be adverse to the interests of the particular restricted society, it may, in fact, prove to be the case that the experience of the small society is better than that of the large, and, if so, it will gain a corresponding advantage. Without definite information as to the past sickness experience of a small society, in which due account is taken of the ages of the members, it is impossible to indicate the probable future experience, and definite information as to past history of this detailed character is seldom obtainable. The first object of insurance should be to make the security for the fulfilment of contracts beyond all question. Possibility of gain should not be allowed to outweigh the more serious consequences of loss. It thus appears to us that, unless there are substantial grounds for believing that a local society will experience a rate of sickness well below the normal standard, preference should be given to a society having a large membership distributed all over the country and in all occupations.

In the second place, it is important that so far as possible the advantages of continuous membership may be obtained. It has been a fairly common practice among Friendly Societies to require a member to relinquish his membership when he ceases to reside in the district within which it operates. The power of expulsion, if contained in the rules of an approved society, may be exercised in a similar manner, and this will militate against a local society, no matter how bright its prospects in the matter of sickness experience, if there is any serious possibility of removal in the future. The insured person so expelled might find it difficult to gain admittance to a local society in the district to which he removes, and if unable to find a new society willing to take him—as will assuredly be the case if he is in bad health—he will have to become a deposit contributor with all the attendant disabilities of that class. In this respect a society with an organisation so wide that it is capable of transferring its members from any one town or village to any other in the United Kingdom, without ever having to require his expulsion, is, in our opinion, in a particularly favourable position to afford facilities to its members.

A third point of considerable importance in choosing a society is that of efficient administration. Insurance in all its branches demands technical and expert treatment. National Health Insurance is no exception to this rule, and very few except those with insurance training can hope to undertake the work with guaranteed success from the outset. The Act is in itself a most complicated measure, and the forms and accounts that will have to be kept by every approved society are, if possible, more intricate than the Act itself. It follows, therefore, that a society which is able to offer to any one of our readers the advantage of existing expert knowledge of insurance and organisation is in the best position to fulfil its duties as an approved society.

As fulfilling all these conditions we notice in the list of societies to which we have already referred the name of the Nurses' Insurance Society. Although this Society by its rules, so we understand, restricts its membership to trained female nurses, it will have a sufficiently wide numerical basis and a sufficiently widely distributed membership to enable it to secure a workable average. Besides this the fact that it has been formed as a separate section of the Royal National Pension Fund for Nurses is in itself a guarantee that the benefits will be wisely and economically administrated and expulsion on account of removal will not be required. The offices of the Society are at 15 Buckingham Street, Strand, London, W.C.

A large number of other societies transacting general business both for men and women under the Act might be mentioned, but space will only permit us to indicate the most prominent of them and those best known, and we do not wish it to be thought that we are making any invidious distinctions. For this reason we merely mention the name of the societies as registered, leaving it to our readers to find a local representative to furnish further information if desired: Ancient Order of Foresters Friendly Society, Hearts of Oak Benefit Society, Liverpool Victoria Approved Society, Manchester Unity of Oddfellows Friendly Society, National Amalgamated Approved Society, National Deposit Friendly Society, Prudential Approved Society.

### Answers to Correspondents.

BENEFIT SOCIETY FOR DOMESTIC SERVANTS.

Miss H. H. D., Bath.—The Act includes under its operations "all persons of either sex, whether British subjects or not, who are engaged in any of the employments specified," domestic service being one of them, so that foreign servants must be insured, though they do not obtain full benefits. At present the societies most suitable for the insurance of domestic servants are: The Prudential Approved Society for Women, the National Amalgamated Approved Society, the Domestic Workers' Union of Great Britain, the Hearts of Oak Benefit Society, the Manchester Unity of Oddfellows Friendly Society and the National Deposit Friendly Society. We are interested to know that you have been a subscriber so many years and are very glad to be of service.

NOTE:—Questions and Correspondence on all doubtful points are invited, and should be addressed to the Editor, The Hospital, 28 Southampton Street, Strand, W.C., and marked "Insurance."

